# Cancer Stem Cells, EMT, and Developmental Pathway Activation in Pancreatic Tumors

**DOI:** 10.3390/cancers4040989

**Published:** 2012-10-12

**Authors:** Sanne Hindriksen, Maarten F. Bijlsma

**Affiliations:** Laboratory for Experimental Oncology and Radiobiology, Academic Medical Centre, Meibergdreef 9, 1105AZ Amsterdam, The Netherlands

**Keywords:** pancreatic cancer, cancer stem cells, epithelial-to-mesenchymal transition, developmental pathways, tumor micro-environment, chemoresistance

## Abstract

Pancreatic cancer is a disease with remarkably poor patient survival rates. The frequent presence of metastases and profound chemoresistance pose a severe problem for the treatment of these tumors. Moreover, cross-talk between the tumor and the local micro-environment contributes to tumorigenicity, metastasis and chemoresistance. Compared to bulk tumor cells, cancer stem cells (CSC) have reduced sensitivity to chemotherapy. CSC are tumor cells with stem-like features that possess the ability to self-renew, but can also give rise to more differentiated progeny. CSC can be identified based on increased *in vitro* spheroid- or colony formation, enhanced *in vivo* tumor initiating potential, or expression of cell surface markers. Since CSC are thought to be required for the maintenance of a tumor cell population, these cells could possibly serve as a therapeutic target. There appears to be a causal relationship between CSC and epithelial-to-mesenchymal transition (EMT) in pancreatic tumors. The occurrence of EMT in pancreatic cancer cells is often accompanied by re-activation of developmental pathways, such as the Hedgehog, WNT, NOTCH, and Nodal/Activin pathways. Therapeutics based on CSC markers, EMT, developmental pathways, or tumor micro-environment could potentially be used to target pancreatic CSC. This may lead to a reduction of tumor growth, metastatic events, and chemoresistance in pancreatic cancer.

## 1. Introduction

### 1.1. Incidence and Mortality of Pancreatic Cancer

Pancreatic cancer is currently one of the most lethal types of cancer. It is estimated that 44,030 new cases of pancreatic cancer arose in the United States in 2011 [1], and 216,000 new cases worldwide in the year 2000 [2]. By far, the most common subtype of pancreatic cancer is pancreatic adenocarcinoma of ductal origin [3] which is thought to be derived from pancreatic intraepithelial neoplasia (PanIN) precursor lesions [4]. What makes pancreatic cancer such a devastating disease is that the survival rates are remarkably poor; approximately 95% of patients die within the first five years after diagnosis [1]. The high mortality rate has been attributed to late detection of the disease due to the absence of early symptoms [5], which is the reason pancreatic cancer is sometimes referred to as the “silent killer”.

An important factor in the fatality of pancreatic cancer is that in the majority of patients the pancreatic cancer cells have already disseminated to other sites in the body at the time of diagnosis [6]. Computational models based on clinical data show that the probability of metastatic disease at moment of diagnosis is so high that aggressive systemic therapy to reduce proliferation of primary and metastatic tumors should be initiated directly after diagnosis, irrespective of distinct evidence for dissemination [6].

### 1.2. Onset of Metastasis Formation

It has been unclear whether the high prevalence of metastasis at the time of diagnosis is due to early metastatic dissemination or if it is the result of late detection of the disease. Sequencing studies using patient material have found that the majority of genetic mutations are shared between metastases and the respective primary pancreatic tumor, which supports a model where metastatic dissemination occurs at a late stage of cancer development [7,8]. In contrast, *in vivo* lineage tracing using a pancreatic ductal adenocarcinoma (PDAC) mouse model indicates that dissemination of pancreatic cancer cells occurs at an early stage of the disease [9]. The apparent contradiction could be explained by differences in mutation and proliferation rates at different stages of cancer progression that may flaw the interpretation of comparison of genetic alterations [9]. On the other hand, the detection and seeding of circulating pancreatic cancer cells in an early stage does not directly prove that these cells can give rise to metastases and may thereby underestimate the time it takes to form metastases [9]. Moreover, the genetically engineered mouse model may not provide a reliable representation of the events occurring in human pancreatic tumors. Establishing the time of dissemination, however, is clinically important, as late formation of metastasis would leave a wider time-window for more effective treatment of pancreatic cancer [7].

## 2. Mutations that Drive Pancreatic Cancer

Genetic analysis of pancreatic tumors has revealed 12 cellular signaling pathways that are frequently altered pancreatic cancer through mutations in at least one of the genes that encode pathway components [10]. These pathways include apoptosis, DNA damage control, regulation of G1/S phase transition, Hedgehog signaling, homophilic cell adhesion, integrin signaling, c-Jun *N*-terminal kinase signaling, KRAS signaling, regulation of invasion, small GTPase-dependant signaling, TGF-β signaling, and WNT/NOTCH signaling [10]. A number of well-described common driver mutations will be discussed in more detail below.

### 2.1. KRAS

The RAS protein family, which consists of HRAS, NRAS, and KRAS, regulates several downstream signaling pathways. Point mutations in the *KRAS* proto-oncogene occur in 83–100% of pancreatic tumors [11,12]. The RAS protein is only active when bound to GTP, which it hydrolyses to GDP through intrinsic GTPase activity [13,14,15]. Exchange of GDP for GTP re-activates the protein [16]. Under normal circumstances, RAS activity is regulated by signaling through upstream receptor tyrosine kinases [17]. However, genetic mutations in the *RAS* gene can give rise to a constitutively active RAS protein with tumorigenic potential [18]. Oncogenic RAS activation has a number of effects, including proliferation of tumor cells, resistance to apoptosis, promotion of tumor cell invasiveness and induction of angiogenesis [19,20,21].

Since KRAS is frequently mutated in pancreatic cancer, inhibitors of RAS and associated signaling pathways are being investigated for treatment purposes. One attempt has been made by targeting RAS via inhibition of farnesylation, a post-translational modification which is required for RAS functionality. Although treatment with a farnesyl transferase inhibitor was well tolerated, addition to standard pancreatic cancer therapy did not appear beneficial compared to standard treatment alone in phase III clinical trials [22]. In a different approach, RNA interference against mutant KRAS has been proven effective in pancreatic cancer cell lines [23]. The RAF/MAPK cascade is a signaling pathway that is activated by RAS. The RAF inhibitor Sorafenib exerts anti-cancer effects in pancreatic cancer cell lines [24] and is currently being tested in multiple clinical trials both alone and in combination with adjuvant therapy. Inhibition of the PI3K/AKT/mTOR signaling axis downstream of RAS using the mTOR inhibiting compounds everolimus and temsirolimus has also given promising results in pancreatic cancer cells [25], and in xenograft mouse model of pancreatic cancer [26], but activity in patients appears minimal [27,28].

More than half of the pancreatic tumors express EGFR [29], a receptor tyrosine kinase that is responsible for activation of RAS [17]. Inhibition of EGFR signaling by treatment with cetuximab in combination with adjuvant therapy seemed a promising approach for pancreatic cancer treatment in phase II clinical trials [30], but showed no benefit over standard treatment in phase III trials [31]. However, addition of the receptor tyrosine kinase inhibitor erlotinib to chemotherapy showed improved overall survival in patients with advanced pancreatic cancer [32]. In the United States, the use of erlotinib in combination with adjuvant therapy has been FDA approved for the treatment of patients with locally advanced, metastatic, or unresectable pancreatic cancer.

### 2.2. P53

P53 is encoded by the *TP53* gene and is present at overall low basal levels as it is targeted for rapid degradation by interaction with the ubiquitin ligase double minute 2 protein (MDM2) [33,34]. Upon DNA damage p53 dissociates from MDM2 leading to increased stability of the protein [34,35]. The accumulation of p53 results in activation of p21 and initiates cell cycle arrest or apoptosis, thereby preventing establishment of genetic lesions [36]. Loss of p53 functionality thus increases mutation rates and can give rise to cancer development [37].

Abnormalities in p53 were found in approximately 60% of pancreatic cancers [38] and in 76% of pancreatic adenocarcinomas [39]. Using a genetically engineered mouse model it has been found that mutant p53 is responsible for rapid development of precursor lesions with activated KRAS into a PDAC and facilitates resistance to mutation induced senescence or apoptosis [40]. Moreover, it was found that tumor cells harboring a point mutation in p53, but not p53 deletion, have a strong predisposition to metastasize, indicating that mutant p53 has an additional function in dissemination of malignant cells [40].

### 2.3. P16INK4A

Entry into the S-phase of the cell cycle is mediated by multiple cyclin-dependant kinases (CDKs) whose activity is regulated via interaction with cyclins [41]. CDK activity mediates phosphorylation of RB protein which allows for E2F dependent transcription and facilitates G1/S phase transition [41,42]. This process can be counteracted by the activity of p16INK4A, which is transcribed from the *INK4A* gene. The protein binds to the cyclin D dependent kinases CDK4 and CDK6, thereby inhibiting their activity leading to senescence [43,44]. P16INK4A is inactivated in 98% of pancreatic carcinomas [45], thereby losing its capacity to inhibit S-phase entry. Inherited mutations in p16INK4A were shown to cause a predisposition to development of pancreatic cancer [46].

Interestingly, the *INK4A* gene overlaps with the *ARF* gene coding for p19ARF protein that inhibits cell cycle progression, in this case via p53 activation [47]. A mouse model has shown that loss of p16INK4A is sufficient to increase the incidence of tumorigenesis, even when p19ARF function is still intact [48]. This indicates that loss of p19ARF is not a prerequisite for cancer development. In line with this finding, the majority of cancer-related mutations in the *INK4A* gene do not disrupt p19ARF integrity [49].

### 2.4. SMAD4

The tumor suppressor gene *SMAD4* encodes a transcription factor that is activated through signaling by members of the transforming growth factor-β (TGF-β) superfamily, including TGF-β1, Activin, and BMP [50,51,52]. Upon stimulation with these cytokines, receptor associated SMADs are phosphorylated, synergize with SMAD4, and translocate to the nucleus where they modulate gene transcription [52,53,54,55,56]. The TGF-β/SMAD pathway affects various events including proliferation, differentiation, migration and apoptosis [57,58,59].

Although overall *SMAD4* mutations occur at low frequency among cancers, alterations in the *SMAD4* gene were found in 48% of pancreatic carcinomas [60]. In a genetically engineered mouse model, *SMAD4* mutations were shown to stimulate the progression of KRAS activated precursor lesions to pancreatic malignancies, indicating that SMAD4 function can inhibit the oncogenic progression of neoplasms [61]. Moreover, inactivation of SMAD4 was associated with poorer prognosis after surgical treatment among patients with pancreatic adenocarcinoma [62].

## 3. Treatment of Pancreatic Cancer

### 3.1. Tumor Resection

Currently, the only potentially curative treatment of pancreatic cancer is surgical resection [63]. A pancreatoduodenectomy referred to as the Whipple procedure [64,65] or alternatively a pylorus-preserving Whipple procedure are the most frequently performed operations for the purpose of removing pancreatic tumors [66]. However, several problems are associated with surgical removal. First, resection of pancreatic carcinomas is obstructed by advanced local tumor extension. Spreading into surrounding tissues often renders complete resection technically challenging or impossible. For example, pancreatic tumors that have invaded the stomach or colon are classified as irresectable [67]. Encasement or invasion of important blood vessels in particular impedes resection of local tumors [67,68]. For this reason, the tumor may be only partially resected at times [69].

Secondly, due to the high frequency of metastatic disease upon diagnosis, resection of the pancreatic tumor is often inadequate for curing pancreatic cancer. In cases where the initial tumor is removed but metastatic cancer cells are still present, patients are likely to die from metastatic disease [70,71]. In most cases, tumor resection in combination with local lymph node dissection will be performed in lymph node positive pancreatic cancer patients, although involvement of lymph nodes is correlated with shorter survival [71,72]. The presence of distant metastatic disease is often viewed as a contra-indication for surgery, although removal of the primary pancreatic tumor in combination with metastasectomy may be beneficial in certain cases [73]. The absence of indications for metastasis does not guarantee that removal of the primary tumor will be effective. Even if evidence for cancer dissemination is lacking at the time of surgery, regional or distant recurrence frequently occurs at a later stage [74,75].

Taking these two factors together, only 10–20% of the patients are eligible for resection of the pancreatic tumor [76,77,78]. In cases where it is possible to surgically remove the complete pancreatic tumor, the resection specimen is microscopically inspected for the presence of cancer cells at the tumor margin [69]. If no malignant cells are observed at the edge of the removed tissue the resection margin is classified as R0, which suggests that all tumor cells have been successfully removed [69]. However, the presence of cancer cells at the border of the removed tissue, designated R1 resection, indicates that there may be residual tumor cells in the body, which reduces the median survival from 16.9 months for R0 resections to 10.9 months for patients with R1 margins [69]. Positive resection margins are found in ~20% of pancreatic cancer patients that undergo surgery [69].

Overall, surgical treatment of pancreatic cancer patients increases the 5 year survival rate to 23%, compared to 5% for untreated patients [76]. These numbers indicate that the benefit from tumor removal is limited, and the vast majority of patients that undergo resection will still succumb to the disease within the 5 year time period. Current trials focus on the benefit of combinations of surgical treatment and adjuvant therapy to improve clinical outcomes [79].

### 3.2. Chemotherapy

Chemotherapy, either in combination with tumor irradiation or surgery or not, is often applied for treatment of pancreatic cancer. The most commonly used chemotherapeutics are gemcitabine and 5-fluorouracil (5-FU). Both chemotherapeutics inhibit DNA synthesis and thereby induce apoptosis [80,81,82,83]. The antimetabolite 5-FU has been developed for the treatment of cancer in 1957 [84,85], and has since been used as a therapeutic for pancreatic cancer despite the marginal efficacy of this agent [86]. Even combined administration with leucovorin, an adjuvant with synergistic activity, did not yield desired responses [87].

In 1997 a paper was published that showed that the nucleoside analogue gemcitabine has an advantage over 5-FU with respect to patient survival and alleviation of symptoms [88]. Gemcitabine is now the most widely used chemotherapeutic for pancreatic carcinomas, though still only 1 in 4 patients benefit from gemcitabine administration, and the median survival period after diagnosis remains less than 6 months [88]. Besides the anti-cancer effects of gemcitabine as a single agent, the compound sensitizes pancreatic cancer cells to radiotherapy [89]. The efficacy of combining gemcitabine with 5-FU or other agents such as cisplatin has also been investigated in phase II and III clinical trials, but these often failed to prove survival benefit [90,91]. In contrast, a large scale phase III clinical study showed that a combination of gemcitabine with capecitabine yielded slightly higher response rates and increased overall survival in comparison to treatment with gemcitabine alone [92]. Moreover, a combination of oxaliplatin, irinotecan, leucovorin, and fluorouracil (FOLFIRINOX) showed improved efficacy compared to gemcitabine therapy in a phase II/III trial of patients with metastatic pancreatic cancer, as this treatment yielded much better response rates, and significantly increased overall- and progression-free survival [93]. However, FOLFIRINOX treatment is associated with profound toxicity, and is thus only suitable for patients with a good performance status [93].

Chemoresistance, either present before the initiation of therapy or acquired at a later stage, is one of the major reasons of treatment failure in pancreatic cancer. Only 24% of patients respond to gemcitabine treatment, and a clinical response to 5-FU occurs in only 5% of pancreatic cancer patients [88]. The exact mechanisms underlying the low sensitivity of pancreatic tumors to chemotherapy are not yet fully elucidated, though studies indicate that interplay between tumor- and stromal cells contributes to resistance to chemotherapy, and that there is a particular role for a specific subpopulation of tumor cells, referred to as cancer stem cells (CSC), in chemoresistance.

## 4. Tumor Micro-Environment

Pancreatic tumors are characterized by an abundance of stromal components, which include fibroblasts, stellate cells, inflammatory cells, blood- and lymphatic-vessels, and extracellular matrix. The role of this desmoplastic reaction in pancreatic cancer is becoming increasingly evident. Inflammation of the pancreas, as well as cross-talk between cancer cells and stellate cells or fibroblasts, contribute to tumor development, growth, dissemination and chemoresistance. Moreover, it is believed that poor vascularization of pancreatic tumors can mediate resistance to chemotherapy.

### 4.1. Pancreatitis

Involvement of tumor micro-environment in induction of cancer is supported by the notion that chronic inflammation of the pancreas is a major risk factor for the development of pancreatic cancer [94]. A number of inflammatory factors found in chronically inflamed pancreases are also present in pancreatic tumors [95]. The influence of inflammation on the promotion of cancer is further illustrated by the finding that in adult mice chronic pancreatitis is a pre-requisite for the development of PDAC from cells expressing oncogenic Kras [96]. The proposed mechanism behind this process is that inflammation of the pancreas inhibits oncogene induced senescence and thereby facilitates the progression of cells harboring mutated Kras to pancreatic cancer cells [97].

Conversely, pancreatic tumor cells also contribute to a local inflammatory response. Granulocytes are attracted to the tumor site via expression of intercellular adhesion molecule-1 (ICAM-1) [98]. Recruitment of tumor associated macrophages is mediated by vascular endothelial growth factor (VEGF), expressed by pancreatic cancer cells [99]. Notably, the mobilization of macrophages stimulates tumor invasion and has accordingly been linked to metastatic disease [100,101]. Tumor infiltrating mast cells were shown to support pancreatic cancer by increasing proliferation, migration, and invasion of pancreatic cancer cells, as well as angiogenesis, and may thereby also promote metastasis [102,103]. In patients, the accumulation of mast cells in pancreatic adenocarcinomas is related with a worse prognosis [103].

Together, these data support a model where cells of the immune system stimulate tumor development, growth, and dissemination, while in turn pancreatic cancer cells promote local inflammation by recruitment of immune cells. The aggressive nature of pancreatic cancer may thus be mediated by crosstalk between inflammatory cells and tumor cells.

### 4.2. Pancreatic Stellate Cells and Fibroblasts

Fibrosis, which is a distinct feature of pancreatic tumors, is at least in part supported by pancreatic stellate cells (PSC). During pancreatitis, factors secreted by cancer cells from the pancreas activate PSC, which in turn start producing the extracellular matrix (ECM) components fibronectin and collagen [104,105,106]. Besides ECM synthesis, secretion of matrix metalloproteinases (MMPs), especially MMP-2 and MMP-9, by activated PSC can also mediate ECM turnover and is thereby believed to promote metastasis [107,108]. PSC autocrine signaling initiates positive feedback loops between pro-inflammatory cytokines [105,109], which contributes to further activation and proliferation of PSC [110].

The Hedgehog signaling pathway also appears to play a role in desmoplasia and tumor outgrowth. Activity of the pro-inflammatory nuclear factor-κB (NF-κB) induces expression of Sonic Hedgehog (SHH) by pancreatic cancer cells and stromal cells, leading to activation of the Hedgehog pathway [111,112]. Moreover, Hedgehog pathway activation may be the result of genetic alteration of components in the pathway [10] or can be induced by KRAS or TGF-β signaling [113,114]. Interestingly, mutant KRAS stimulates Hedgehog production in pancreatic tumor cells, but seems to reduce their responsiveness to this ligand. This provides a possible mechanism for the paracrine signaling seen for Hedgehog in PDAC [115]. SHH signaling mediates activation of PSC, fibroblast infiltration, and increased secretion of fibronectin, collagen type I, MMPs, and TGF-β1 [116,117]. Furthermore, expression of SHH promotes proliferation, invasion, and metastasis of pancreatic cancer cells [111,112,116,118].

Through activation by pancreatic cancer cells, PCS also secrete the ECM component periostin into the tumor micro-environment [119,120]. Periostin acts as a ligand of the β4-integrin receptor on pancreatic cancer cells and activates downstream AKT and MAPK pathways [119,120]. Activation of these pathways by PSC signaling supports oncogenic progression and metastasis by promoting tumor growth, survival, and invasion under hypoxic and nutrient deprived conditions [119,120,121,122].

PSC can also enhance the invasion-promoting effect of SERPINE2, which is secreted by pancreatic tumor cells [123]. Moreover, SERPINE2 does not affect tumor growth in the absence of PSC, but augments proliferation of pancreatic cancer cells and thus tumor growth only in the presence of PSC [123]. High SERPINE2 expression is accompanied by increased deposition of ECM [123,124].

Thus, not only inflammatory cells but also other stromal components such as fibroblasts, PCS, and ECM support pancreatic tumor progression. The interactions of tumor cells and their micro-environment are fairly complex, with many signaling pathways involved.

### 4.3. Stromal Factors Can Mediate Chemoresistance

Tumor stroma is not only thought to play a role in cancer development and maintenance, but also in resistance to chemotherapy. Factors secreted by PSC inhibit the effect of gemcitabine on pancreatic cancer cell lines [121]. Chemoresistance is partly mediated by ECM components, as the ECM proteins fibronectin, laminin, collagen type I, and collagen type IV support cancer cell proliferation and resistance to various cytotoxic agents, except to gemcitabine [125]. Furthermore, periostin was shown to decrease sensitivity to chemotherapy with gemcitabine or 5-FU through signaling via collagen type I [119].

In another example, production of nitric oxide by fibroblasts mediates IL-1β and NF-κB dependent chemoresistance of pancreatic tumor cells [126,127]. Through inhibition of NF-κB, these cancer cells are re-sensitized to chemotherapy induced apoptosis [128,129]. Apoptosis of tumor cells is mediated by the pro-apoptotic factor BAX and the anti-apoptotic Bcl-2 and Bcl-xL. As such, expression levels of these apoptotic factors are predictive of the chemoresponsiveness of pancreatic tumors [130,131].

As for pancreatic tumor growth and invasion, there are various stromal components that can promote chemoresistance in pancreatic tumor cells. Several pathways appear to play a role in sensitivity to chemotherapy, so tumor-stroma interactions may contribute to resistance via multiple mechanisms.

### 4.4. Tumor Vascularization

Proteins that are secreted by stromal cells promote tumor vascularization. VEGF and MMP-9 have been identified as factors that stimulate pancreatic tumor angiogenesis [132,133,134]. Several studies have found a correlation between pro-angiogenic factors or intratumoral microvessel density and metastasis [135,136,137]. Cancer cell dissemination is increased in hypervascularized tumors, and extensive tumor vascularization is hence a hallmark of poor prognosis [135,137].

In fact, due to the abundance of stroma in pancreatic cancer, vascularization of the tumor is reduced compared with the normal pancreas [138]. PSC hinder angiogenesis in pancreatic tumors by secreting ECM components leading to fibrosis and by promoting secretion of the anti-angiogenic factor endostatin from cancer cells [139]. Poor vascularization has been implicated to contribute to chemoresistance as it hampers delivery of chemotherapeutic agents to the target site. In a genetically engineered mouse model inhibition of Hedgehog signaling disrupted tumor stroma and thereby increased intratumoral microvessel density [140]. This resulted in enhanced delivery of gemcitabine to the pancreatic tumor and stalled tumor progression in previously chemoresistant tumors [140]. Furthermore, depletion of the ECM component hyaluronan expanded blood vessels and facilitated increased intratumoral delivery of gemcitabine in a genetically engineered mouse model of PDAC, thereby inhibiting tumor growth and improving survival [141].

## 5. Cancer Stem Cells in Pancreatic Cancer

### 5.1. The Cancer Stem Cell Concept

Cancer cells within a tumor are heterogeneous [142], indicating that some cancer cells may have slightly different properties than others. A concept has been proposed stating that a specific subpopulation of cancer cells with stem cell-like properties are responsible for tumor growth, while other cancer cells do not contribute to tumor expansion. The tumor initiating cells, or cancer stem cells, are defined as cancer cells with two key features. First of all, CSC should be able to self-renew, meaning that they maintain their stem cell characteristics during cell division [143]. Secondly, they can give rise to more differentiated progeny, and are required to replenish the population of non-CSC [143]. As CSC are thought to be responsible for maintaining the tumor cell population, eradication of these cells should be sufficient, and essential, to stall tumor progression. However, it is believed that pancreatic CSC are more resistant to chemotherapy than more differentiated cells.

### 5.2. Identification of Cancer Stem Cells

In order to define a subset of cancer cells that are capable of self-renewal and generate a heterogeneous cancer cell population, a number of functional assays are employed. The use of mouse xenograft transplantations has become the gold standard to test a cell population for CSC characteristics [143,144]. Cells expressing stem cell markers can be isolated by fluorescence-activated cell sorting (FACS) [144], and subsequent engraftment of a specific subset of cells in immunocompromised mice allows evaluation of the tumor initiating potential of these cells. Tumor regeneration can be evaluated by serial transplantation, and the xenograft tumor that is derived from these cells can subsequently be tested for the presence of a heterogeneous cell population.

A cell population can be enriched for cancer stem cells *in vitro* by culturing them under specific conditions that allow for outgrowth of undifferentiated cells. This can be achieved by cultivation in serum-free medium with addition of growth factors and other supplements [145]. It is believed that the resulting spheroids of cells are enriched in CSC. In addition, colony formation assays may be used to determine outgrowth potential of subsets of cancer cells *in vitro*. Dye exclusion is sometimes used as a characteristic to identify a CSC rich side population, although this feature may not be restricted to CSC and in certain tissues CSC will not be included in the side population [143].

Support for the presence of CSC has been found in various types of cancer, including blood-, breast-, and brain cancer [146,147,148]. A number of CSC surface markers identified in these studies have been investigated for their significance in pancreatic cancer cells with stem cell-like characteristics, which will be discussed in the following section. An overview of the correlations between individual pancreatic CSC markers, dye exclusion, CSC specific miRNAs, and functional parameters is provided in Table 1.

**Table 1:** Correlations between expression of stem cell markers, dye exclusion, CSC specific miRNAs, and *in vivo* tumorigenicity. Green: Partial overlap between characteristics. Red: No significantly increased overlap between characteristics. Grey: No data available. Reference numbers are shown for each correlation.

	CD44/CD24/ESA	CD133	ALDH	c-MET	dye exclusion	miRNA	functional assay
CD44/CD24/ESA	n/a	154	159 160	161	–	169	149 150 160
CD133	154	n/a	159	161	162 163	169	154
ALDH	159 160	159	n/a	161	–	–	159 160
c-MET	161	161	161	n/a	–	–	161
dye exclusion	–	162 163	–	–	n/a	–	162 163
miRNA	169	169	–	–	–	n/a	168 169
functional assay	149 150 160	154	159 160	161	162 163	168 169	n/a

### 5.3. CD44, CD24, and ESA

In one study, primary human pancreatic adenocarcinomas were grown in the immunocompromised NOD/SCID mice. From these xenograft tumors, cells were separated into different subpopulations based on the expression of CD44, CD24, ESA, or combinations of these markers. Compared to an unsorted cell population, cells expressing one or multiple markers exhibited increased tumorigenicity upon injection into NOD/SCID mice, and the tumorigenic potential increased with the number of different markers that were expressed [149]. Enhanced tumorigenicity of marker expressing cells was confirmed by orthotopic implantation of CD44^+^CD24^+^ESA^+^ versus CD44^−^CD24^−^ESA^−^ cells in NOD/SCID mice, with tumor forming potential being 100 fold higher in CD44^+^CD24^+^ESA^+^ cells [149]. In agreement with the CSC definition, CD44^+^CD24^+^ESA^+^ cells were able to give rise to a heterogeneous tumor cell population, with the expression patterns of CD44, CD24, and ESA resembling that of the primary tumor [149]. Notably, the CD44^+^CD24^+^ESA^+^ cells represent a particularly small cell fraction in human pancreatic adenocarcinomas, as this subset comprises less than 1% of the pancreatic cancer cell population [149].

Using the pancreatic cancer cell line PANC-1, it was also found that CD44^+^CD24^+^ cells have a higher *in vivo* tumorigenic potential than cells expressing either marker, which are in turn more tumorigenic than CD44^−^CD24^−^ cells [150]. For this cell line, CD44^+^CD24^+^ cells were enriched under culturing conditions that allow for spheroid formation [151]. CD44^+^CD24^+^ cells isolated from the PANC-1 cell line had high expression of the embryonic stem-cell related genes *OCT4* and *NANOG* [152], providing another link between this cell population and stemness as defined by markers from developmental biology.

Since many supposed markers of CSC are glycoproteins, the glycosylation patterns of supposed pancreatic CSC was investigated by lectin microarray. Because nearly all PANC-1 cells were found positive for CD44, cells were sorted into CD44^+^CD24^+^ and CD44^+^CD24^−^ populations. Increased fucosylation and galactosylation of glycoproteins was found in the CD44^+^CD24^+^ population, and 17 differentially expressed glycoproteins were identified by LC-MS/MS [153].

### 5.4. CD133

CD133 is another surface protein that is used as a marker for CSC. Interestingly, CD133 was hardly expressed in normal tissue of the pancreas, whereas 1.8% of pancreatic tumor were classified as CD133 positive [154]. 5 × 10^2^ CD133^+^ cells isolated from human pancreatic tumors resuspended in matrigel were able to generate tumors in athymic mice, a feature that was not observed for CD133^−^ cells even when up to 10^6^ were engrafted [154]. The tumor generated by transplantation of CD133 positive cells was morphologically identical to the primary tumor, indicating that the CD133^+^ cells can generate a heterogeneous cell population [154]. Serial transplantation of CD133^+^ cells up to three generations further underlines the tumor regenerating capacity of these cells [154]. Another indication that CD133 positive cells are more tumorigenic is that the percentage of CD133^+^ cells in the pancreatic tumor correlated directly with *in vivo* xenograft outgrowth upon transplantation of unsorted cells [154].

The pancreatic cancer cell line L3.6pl contains a subset of CD133 expressing cells. 10^3^ of these CD133^+^ cells are sufficient to form a tumor upon implantation in the pancreas of athymic mice, while 10^6^ CD133^−^ cells did not give rise to a tumor [154]. Moreover, CD133^+^ L3.6lp cells formed spheroids when cultivated in serum-free medium, but CD133^−^ cells were unable to survive under these conditions [154].

In contrast to the findings described above, other studies do not support the link between CD133 expression and pancreatic CSC. Using an antibody against the AC133 epitope that is generally associated with stem- and progenitor-cells, CD133 was found to be expressed on the apical membrane in the majority of ductal cells in normal pancreas and PDAC [155,156]. AC133 staining also increased with time in culture, potentially due to better survival of ductal cells compared to other cell types or transdifferentiation of cells *in vitro* [156]. Interestingly, cells with cytoplasmic AC133 staining occurred in both non-malignant pancreatic tissue and pancreatic tumors, though at very low frequency [155]. The authors speculated that these non-ductal pancreatic cells that have cytoplasmic CD133 expression could be potential candidates as stem cells or CSC [155]. Also, CD44^+^CD133^+^ cells that reside mainly at the centroacinar region are put forward as an interesting subpopulation when attempting to identify pancreatic CSC [157].

The discrepancies between the studies on CD133 expression in the pancreas could have many reasons. For one, the method of tissue preparation prior to immunostaining was shown to influence the results [156]. Also, the type of CD133 antibody that is used can be important, as antibodies targeting different epitopes that may be subject to splicing or posttranslational modifications could yield completely different results [155,158]. The sensitivity of signal detection may also be an issue. For example, the AC133 antibody was found particularly difficult to detect, so antibody concentration and detection method are important for the assessment of CD133 expression [155].

### 5.5. Aldehyde Dehydrogenase

Expression of aldehyde dehydrogenase (ALDH) is heterogeneous both among and within patient samples of PDAC [159]. Implantation of 100 ALDH^high^ cells isolated from human pancreatic adenocarcinoma derived xenografts gave rise to a tumor in NOD/SCID mice in 70% of cases, whereas transplantation of 100 ALDH^low^ cells did not result in tumor formation [159]. Notably, the capability of ALDH^high^ or ALDH^low^ cells to initiate tumors upon transplantation was irrespective of CD133 status [159]. The tumors originating from xenografted ALDH^high^ cells contained both ALDH^high^ and ALDH^low^ cells, indicating that these cells can generate a heterogeneous cell population [159].

An ALDH^high^ subpopulation of cells is present in both the L3.6pl and CAPAN-1 pancreatic cancer cell lines [159,160]. Implantation of ALDH^high^ cells of each cell line in NOD/SCID mice lead to increased formation of tumors compared to transplantation of ALDH^low^ or unsorted cells [159,160]. The L3.6pl xenograft tumors contained small clusters of ALDH^high^ cells, again providing evidence that this population can give rise to a heterogeneous tumor [159]. Serial transplantation of ALDH^high^ CAPAN-1 cells confirmed their self-renewal potential, and these cells also showed increased *in vitro* growth potential compared to ALDH^low^ or unsorted cells [160]. Cultivation of L3.6pl cells under spheroid supporting conditions increased the fraction of ALDH^high^ cells, indicating that ALDH expression may indeed be a marker of undifferentiated cells [159].

To identify if ALDH^high^ pancreatic cancer cells may represent the same subset of cells as the CD44^+^CD24^+^ population, coexpression of these markers has been investigated. In xenografts derived from primary pancreatic tumors the overlap between ALDH^high^ and CD44^+^CD24^+^ cells was very limited [160]. Both ALDH expression and CD44 and CD24 expression were positively correlated with xenograft formation in NOD/SCID mice, with both populations being equally capable of tumor initiation [160]. The small population of pancreatic cancer cells that expressed ALDH, CD44, and CD24 exhibited the highest tumorigenic potential *in vivo*, although the differences with cells expressing only ALDH or CD44 and CD24 were not statistically significant [160].

### 5.6. c-MET

As c-MET is a putative stem cell marker of the pancreas and increased expression had previously been linked with pancreatic adenocarcinoma, the potential of this receptor as a marker of pancreatic CSC was investigated. High expression of c-MET partially overlapped with expression of the surface markers CD44, CD24, ESA, CD133 and ALDH. c-MET^high^ cells isolated from pancreatic xenograft tumors showed increased tumorigenic potential in NOD/SCID mice compared to c-MET^low^ or c-MET^−^ cells [161]. c-MET appeared to be a more suitable marker than CD44 or CD133 expression, though CD44^+^CD24^+^ESA^+^ cells were more tumorigenic than c-MET^high^ cells [161]. c-MET^high^CD44^+^ cells exerted the highest *in vivo* tumorigenicity, generating a tumor in 90% of cases when 1,000 cells suspended in matrigel were injected subcutaneously, compared to 50% tumor formation for c-MET^high^cells [161]. Xenograft tumors derived from c-MET^high^CD44^+^ cells were made up of both marker-positive and negative cells, therein resembling the primary tumor [161].

Besides tumor formation upon injection in mice, c-MET^high^ cells isolated from these primary tumor xenografts were also capable of forming spheroids *in vitro* whereas c-MET^low^ or c-MET^-^ cells were not [161]. Inhibition or knockdown of c-MET reduced the spheroid forming potential of c-MET^high^ cells and increased apoptosis, indicating that c-MET is not only a marker but also plays a functional role in self-renewal [161]. Furthermore, treatment of mice bearing xenograft pancreatic tumors with a c-MET inhibitor XL184 reduced tumor growth, especially in combination with gemcitabine [161]. In both cases, the c-MET^high^CD44^+^ population of cancer cells was specifically depleted [161]. These findings suggest that c-MET could potentially be used as a target for therapy.

### 5.7. Dye Exclusion

Specific proteins named ABC-transporters are capable of carrying a variety of molecules over the cell membrane. A side population of pancreatic cancer cells can extrude the dye Hoechst 33342 via these ABC-transporters [162,163]. The pancreatic cancer cell lines Capan-2, SW1990, and BxPC-3 each contain around 3% of side population cells [162]. Side population cells isolated from the PANC-1 and SW1990 pancreatic cancer cell lines had a higher *in vitro* proliferation rate than non-side population cells [162,163]. Moreover, the side population cells were able to produce cells that have both side population features and non-side population features in culture, whereas non-side population cells could only generate non-side population progeny [162,163]. Pancreatic side population cells from these cell lines also had enhanced *in vivo* tumorigenicity compared to unselected or non-side population cells [162,163]. Notably, cells of the side population had higher CD133 expression than non-side population cells [162,163].

### 5.8. MicroRNAs

Recent evidence suggests a link between the expression of microRNAs (miRNAs) and pancreatic cancer stem cells. MiRNAs regulate gene expression by binding to messenger RNAs (mRNAs) and thereby mediating their degradation [164], decay [165], or repression of translation [166]. A comparison between miRNA expression in pancreatic cancer cell lines cultured as spheroids enriched for CSC and adherent cells revealed a number of CSC specific miRNAs, including miR-99a, miR-100, miR-125b, miR-192, and miR-429 [167]. Furthermore, a high correlation was found between differentially expressed miRNAs and stem cell related mRNAs, indicating that miRNAs may indeed play an important role in the regulation of pathways associated with stemness [167]. More specifically, in pancreatic cancer cells ZEB1 represses miR-203, which is an inhibitor of stemness, and the miR-200 family members miR-141, miR-200a, b, c, and miR-429, which also regulate expression of stem cell factors [168]. Notably, ZEB1 knockdown reduced the tumor initiating potential upon transplantation in nude mice [168]. In another example the expression of the p53 regulated miR-34, which acts as a downregulator of the NOTCH and Bcl-2 pathways, is reduced in the CD44^+^CD133^+^ subpopulation of the pancreatic cancer cell line MiaPaCa2 [169]. Restoration of the miR-34 levels reduced the proportion of CSC in the population, and simultaneously diminished *in vitro* spheroid growth and *in vivo* tumor formation [169].

## 6. Developmental Pathways in Cancer Stem Cell Biology

Several lines of evidence suggest a link between the epithelial-to-mesenchymal transition (EMT) and CSC characteristics in pancreatic cancer. Alterations in genes associated with developmental pathways such as Hedgehog, WNT, and NOTCH is common in pancreatic cancer [10], and could facilitate EMT. The link between activation of developmental pathways leading to EMT and CSC is discussed here.

### 6.1. The Epithelial-to-Mesenchymal Transition

In embryogenesis, the epithelial-to-mesenchymal transition is required for development of the vertebral column [170]. Morphologically, cells lose their apical-basal polarization and adopt front-to-back polarization which is important for motility [171]. Mesenchymal cells can adhere to the ECM using filopodia and pseudopodia in order to migrate away from the epithelium [172,173]. At their target site, mesenchymal cells have the capacity to undergo differentiation [173].

Several molecular markers distinguish mesenchymal cells from epithelial cells and are used to track the EMT. E-cadherin is an adhesion molecule which is lost upon EMT, thereby contributing to the ability to migrate [174]. Snail and Slug belong to a family of zinc finger proteins that mediate downregulation of E-cadherin [175]. ZEB1, Twist, and LEF-1 are also repressors of E-cadherin and these proteins are used as mesenchymal markers as well [176,177,178]. Moreover, high expression of the vimentin intermediate filament is characteristic for mesenchymal cells [179].

EMT is an event that can also occur in tumor cells. EMT is believed to enhance metastasis because of the increased migratory capacity of mesenchymal cells. Accordingly, circulating pancreatic cells underwent EMT prior to dissemination in a genetically engineered mouse model, as identified by expression of the mesenchymal markers ZEB1, Slug, Snail1 and others [9]. The majority of pancreatic cancers are positive for markers indicative of EMT [180].

### 6.2. Hedgehog Signaling

The Hedgehog pathway is primarily known for its role in embryonic development [181,182]. Upon secretion, Sonic Hedgehog binds to a receptor designated Patched, thereby abrogating inhibition of the signal transducer Smoothened [183,184]. Active Smoothened signals via GLI, leading to the expression of Hedgehog responsive genes [185,186].

Aberrant expression of Sonic hedgehog is frequently observed in pancreatic adenocarcinomas as well as PanIN precursor lesions, with increasing expression upon tumor progression [187]. Expression of SHH was accompanied by detection of Patched and Smoothened in human neoplastic pancreases and the surrounding mesenchymal cells [187]. It was found that Hedgehog signaling is required for outgrowth of pancreatic tumors [188]. Accordingly, inhibition of Hedgehog signaling reduced proliferation and mediated apoptosis in pancreatic cancer cell lines both *in vitro* and *in vivo* [187].

Hedgehog signaling also plays a role in EMT. Hedgehog pathway inhibition by treatment with cyclopamine reduced expression of Snail and upregulated E-cadherin in pancreatic cancer cell lines, changes which are characteristic of suppression of EMT [118]. Moreover, the *in vitro* invasiveness and *in vivo* metastatic spread of these cell lines was reduced by Hedgehog inhibition [118]. Conversely, overexpression of SHH increased metastasis in a athymic nude mouse model, an event that could be counteracted by treatment with a SHH neutralizing antibody [189]. It is suggested that besides autocrine effects of SHH secreted by malignant epithelial cells, the regulation of tumor micro-environment, and in particular promotion of angiogenesis, has a more profound influence on metastatic potential [189].

Evidence suggests that expression of SHH is particularly important in CSC of the pancreas. SHH expression was ~46 times higher in CD44^+^CD24^+^ESA^+^ xenografted pancreatic tumor cells than in normal pancreatic epithelial cells, compared to a 4-fold increase of SHH expression in unsorted pancreatic cancer xenograft cells [149]. Hedgehog signaling components were also upregulated in cells of pancreatic cancer cell lines cultivated as CSC supporting spheroids [190]. In addition, these spheroids harbored increased expression of genes that are activated if cooperation between the Hedgehog and EGFR pathways occurs, and depend on these Hedgehog-EGFR cooperation responsive genes for *in vitro* spheroid formation and *in vivo* tumor growth [190]. Inhibition of the Hedgehog pathway with cyclopamine reduced the expression of supposed CSC markers CD44, CD133 and ALDH in pancreatic cancer cell lines [118,191]. (−)-epigallocatechin-3-gallate (EGCG) is a compound without known target, but in cultured pancreatic cancer cells it simultaneously inhibits expression of Hedgehog pathway components, the EMT markers Snail, Slug, and ZEB1, and the pluripotency factors Nanog, c-MYC, and OCT-4 [192]. Moreover, the compound reduces spheroid formation from pancreatic tumor cells, outgrowth of colonies *in vitro*, and induces apoptosis [192]. These data suggest a link between Hedgehog signaling, stem cell characteristics, and EMT in pancreatic cancer.

### 6.3. WNT Signaling

The canonical WNT pathway describes binding of extracellular WNT to Frizzled family cell surface receptors and LRP co-receptors, leading to activation of Dishevelled [193,194]. Dishevelled inhibits activity of the axin/GSK-3/APC complex which mediates proteolytic degradation of β-catenin, resulting in an increase of β-catenin levels [195,196]. Interaction of β-catenin with LEF/TCF transcription factor family members promotes the expression of target genes [197,198]. WNT/β-catenin signaling is important for maintenance of the self-renewal capacity of embryonic stem cells and induces OCT-3/4 dependent upregulation of the stem cell factor Nanog [199]. Conversely, overexpression of OCT-4 increased β-catenin activity and inhibited cell differentiation [200]. Although the WNT/β-catenin pathway is essential for normal development of the pancreas, stabilization of β-catenin at a later stage can give rise to pancreatic tumors [201]. Aberrations in the pathway leading to increased levels of β-catenin are found in 65% of pancreatic adenocarcinomas [202]. Not only cancer development, but also dissemination may be enhanced by signaling via the canonical WNT pathway, as it was shown that WNT/β-catenin/LEF-1 mediated transcription can induce EMT [203].

Besides the canonical WNT/β-catenin pathway, there are at least two non-canonical pathways downstream of WNT. The WNT/calcium pathway signals via heterotrimeric G-protein dependent activation of the phosphatidylinositol pathway, which leads to release of intracellular Ca^2+^ [204]. As a consequence, Ca^2+^ sensitive enzymes including PKC and Ca^2+^/calmodium-dependent kinase (CaMK)II are activated [205,206]. WNT/calcium mediated upregulation of PKC leads to increased cell migration [207]. Moreover, acquisition of mesenchymal morphology, accompanied by upregulation of Snail and loss of E-cadherin, indicate that increased expression of PKC also contributes to EMT [207]. Interestingly, activation of the WNT/calcium pathway also increased expression of the CSC marker CD44 [207].

The Planar Cell Polarity pathway involves a specific set of proteins including VANGL2, CELSR1, SCRB1, and PTK7 [208,209,210]. Activation of Rho family GTPases, including RHO, CDC42, and RAC, leads to activation of downstream Rho kinase and JNK [211,212,213]. The Planar Cell Polarity pathway is involved in polarization and motility via organization of the cytoskeleton [214], again providing a link between WNT signaling and cell migration. However, direct evidence for the activation of the Planar Cell Polarity pathway in pancreatic cancer remains elusive.

Although the relation between WNT signaling and CSC characteristics in pancreatic cancer has not been extensively studied thus far, a well-defined connection has been found in other types of tumors, particularly colon cancer [215]. Further investigation might also identify an important role for the WNT pathway in pancreatic CSC.

### 6.4. NOTCH Signaling

Activation of the NOTCH pathway depends on binding of a Delta-type or Jagged-type ligands expressed on the cell surface to a NOTCH-like receptor on the surface of a neighboring cell [216,217,218]. The interaction between ligand and receptor causes cleavage of NOTCH, releasing the soluble intracellular domain [219]. This intracellular NOTCH fragment translocates to the nucleus, where it associates with CBF1/Su(H)/LAG1 (CSL) family proteins [220]. NOTCH binding switches the activity of CSL DNA binding proteins from transcription repressors to transcription activators to mediate the expression of target genes [220]. Regulation of NOTCH signaling depends on feedback loops that regulate receptor and ligand transcription [221], modulation of receptor and ligand activity via posttranslational modifications [222], or activity of inhibitory factors [223]. Moreover, NOTCH signaling is involved in cross-talk with the WNT signaling pathway. Several nodes of interaction have been identified which mediate inhibitory or activating effects that the NOTCH and WNT pathways exert on each other [224,225,226,227,228,229].

NOTCH signaling plays an important role in regulation of embryonic development [230,231], by regulating both restriction and specification of cell fate [232,233]. The NOTCH pathway is involved in pancreatic organogenesis, through suppression of progenitor cell differentiation and by directing cell fate [234,235]. Moreover, NOTCH is involved in organization of the actin cytoskeleton to facilitate axon extension and acts as a mediator of cell migration [236,237]. Besides its role in the developing pancreas, the NOTCH signaling pathway is also activated in pancreatic precursor lesions and pancreatic cancer, likely due to upregulation of Delta-type or Jagged-type ligands [238,239]. Increased expression of NOTCH pathway components has been associated with EMT, and enhanced migration and invasion of pancreatic cancer cells, whereas inhibition of NOTCH signaling reverted the EMT phenotype and decreased expression of vimentin, Snail, Slug, and ZEB1 in human pancreatic cancer cell lines [240,241]. In turn, it was shown that ZEB1 expression directly controls NOTCH signaling activity [242]. Besides induction of EMT, NOTCH overexpression mediated upregulation of CSC markers CD44 and ESA [241], while NOTCH inhibition depleted ALDH^high^ cells [239]. Moreover, pancreatic CSC identified by CD44 and CD133 expression or dye exclusion that were isolated from cell lines have higher expression levels of NOTCH than non-CSC [163,169].

### 6.5. TGF-β Superfamily: Nodal/Activin Signaling

Nodal and Activin belong to the TGF-β superfamily of secreted growth factors. The proteins are expressed during embryonic development, and are essential for embryonic stem cell maintenance [243,244]. Both Nodal and Activin can bind to the ALK4 and ALK7 receptors, while Cripto-1 functions as a co-receptor only for Nodal [245,246]. Although the molecular mechanisms of Nodal/Activin signaling are not fully understood, it is likely that Nodal signals via SMAD2 [247], while Activin regulates SMAD2 and SMAD3 [51]. Both SMAD2 and SMAD3 interact with SMAD4 [248] and moreover directly control Nanog expression to block stem cell differentiation [249]. Furthermore, Activin appears to activate WNT, FGF, and Nodal pathways while repressing BMP signaling, which could contribute to maintaining stemness [250]. Indeed, whereas predominant Activin signaling and to a lesser extent FGF signaling maintains cells in a pluri- or multipotent state, expression of BMP either or not in combination with Activin or FGF signaling drives cell differentiation and controls cell fate in various tissues, including pancreas [251,252].

In addition to its role in embryonic development, Nodal/Activin signaling was also identified in pancreatic cancer [253,254]. The pathway was shown to be specifically activated in spheroid-derived or CD133^+^ pancreatic CSC from xenograft tumors or pancreatic cancer cell lines [254]. Signaling via Nodal/Activin, but not TGF-β1, is required for the ability of these CSC to undergo self-renewal, as determined by increased spheroid formation capacity upon over-activation of the pathway and reduced spheroid formation as a result of pathway inhibition [254]. Moreover, stimulation of cells with Nodal/Activin increased the invasive potential, a feature which was reverted by suppression of Nodal/Activin pathway activity [254].

Contrary to what might be expected, data suggest that in addition to preventing differentiation, Activin signaling also plays a role in repressing EMT. Inhibition of Activin decreases expression of miRNA-200 family members, which act as ZEB1 repressors, to allow for EMT to proceed [255]. Although the underlying mechanism is unclear, the EMT related transcription factor ZEB2 was shown to antagonise Nodal/Activin signaling through interaction with SMAD proteins [256]. Possibly, the synergistic effect of Activin and BMP to drive cell differentiation [251,252] plays a role in repression of EMT. In case of Nodal/Activin signaling, the relation between EMT and CSC thus appears less straightforward. Nevertheless, the signaling pathway is involved in maintenance of stem-like properties in both embryonic stem cells and CSC.

### 6.6. Developmental Pathways as Drivers of Oncogenesis

The fact that activation of developmental pathways in pancreatic cancer is such a common event suggests that these pathways could be essential for certain pancreatic tumors. As described, signaling via embryonic pathways can induce an epithelial-to-mesenchymal transition in pancreatic cancer cells. This raises the possibility that activation of embryonic signaling pathways could drive pancreatic oncogenesis via induction of an EMT, and may therefore be required for both development and maintenance of certain pancreatic tumors, in addition to the role of EMT in cancer cell dissemination.

This notion is supported by a study that examined the dependency on KRAS signaling in pancreatic cancer cell lines that harbor activating mutations in *KRAS*. Two classes of pancreatic cancer cell lines could be identified, based on their requirement for KRAS signaling to maintain viability. Cell lines dependent on KRAS expression had a predominant epithelial phenotype, and exhibited markers of differentiation. KRAS independent cell lines were less uniform in their epithelial morphology and showed features of EMT, including loss of E-cadherin and expression of vimentin [257]. Moreover, induction of EMT in KRAS dependent cell lines diminished the requirement of KRAS signaling for cell survival [257]. Conversely, reversal of EMT in KRAS independent cell lines made these cells dependent on KRAS signaling [257]. This suggests that EMT may abolish the requirement of oncogene activation for tumor cell survival [257].

## 7. Pancreatic Cancer Stem Cells and EMT

Several lines of evidence indicate that there is a link between CSC and epithelial-to-mesenchymal transition in the pancreas. The relation between these two entities is however not straightforward. It appears that CSC are more prone to undergoing EMT, while conversely EMT could endow cancer cells with stem-like features. It is thus difficult to determine if the generation of CSC or acquisition of EMT occurs first. The relationship between pancreatic CSC and EMT with respect to several events is discussed in this section.

### 7.1. Cancer Stem Cells Are Less Sensitive to Chemotherapy

One important feature of CSC is their increased resistance to chemotherapeutic treatment. Gemcitabine resistant cells derived from pancreatic cancer cell lines exhibited increased *in vitro* clonogenicity and spheroid forming capacity, and enhanced *in vivo* tumorigenicity [258]. *In vitro* generated gemcitabine resistant pancreatic cancer cells were marked by high expression levels of CD44 [191,258], while in another study increased CD24 and ESA expression, as well as activation of the c-MET receptor were also detected in gemcitabine resistant cells [259]. A different experiment showed enrichment for CD24 and ALDH after *in vivo* gemcitabine treatment of pancreatic xenografts [260]. When treatment was ceased, tumor outgrowth was resumed and CSC marker expression returned to pre-treatment levels [260]. Treatment with gemcitabine also increased the proportion of CD133^+^ cells in the tumor cell population both *in vitro* and *in vivo*, and cells positive for CD133 showed strong resistance to gemcitabine induced apoptosis [154,191,261].

The enrichment for gemcitabine resistant CSC is accompanied by the induction of an EMT, as assessed by morphology, mesenchymal marker expression, and increased invasive and migratory potential [151,240,259,262]. A comparison of chemo-sensitive and -resistant pancreatic cancer cell lines suggests that EMT is directly related with treatment response [263,264]. Accordingly, reversal of EMT in resistant cell lines increased sensitivity to chemotoxins, providing evidence that the occurrence of EMT itself is an important event that leads to chemoresistance [263,264].

Besides EMT markers, components of the Hedgehog pathway are also upregulated in gemcitabine resistant cell line derived pancreatic cancer cells [191]. These cells can be resensitized to gemcitabine by treatment with the Hedgehog pathway inhibitor cyclopamine [191]. Furthermore, inhibition of the Hedgehog pathway during gemcitabine treatment can also decrease CSC marker expression and mediate tumor regression in a xenograft model of pancreatic cancer [260]. Tumor stabilization and CSC depletion was also observed when Nodal/Activin signaling was blocked simultaneously with gemcitabine treatment and Hedgehog inhibition [254]. Moreover, it is suggested that activation of the NOTCH pathway is involved in the mesenchymal character of gemcitabine resistant cells [240]. In addition, activation of the FAK, PI3K/AKT, and ERK signaling pathways and enhanced transcription of NF-κB and β-catenin by stimulation of the chemokine receptor CXCR4 could also reduce sensitivity to gemcitabine [265].

A clue to the mechanism of resistance in pancreatic CSC is that the cells are often resistant to various agents, a feature referred to as multidrug resistance [258,263]. Membrane transporters that pump toxic agents out of the cell, the ABC transporters, are thought to be responsible for multidrug resistance [266]. In gemcitabine resistant CSC, ABC transporters, including ABCG2 and ABCB1, are indeed overexpressed and could thus mediate chemoresistance [151,191,258]. Interestingly, Hedgehog pathway inhibition via treatment with cyclopamine downregulates ABCG2 and ABCB1 expression, and restores chemosensitivity [191].

Besides upregulation of ABC transporters, other features of CSC may also contribute to the reduced sensitivity to chemotherapy. For example, there are indications that pancreatic CSC constitute a slow cycling population of cells [267], and therefore may be less affected by chemotherapeutic treatment. Moreover, it is suggested that CSC more efficiently repair DNA-damage after exposure to gemcitabine, which could facilitate evasion of apoptosis [268].

The notion that CSC have decreased sensitivity to chemotherapeutic agents compared to non-CSC suggests that these cells could underlie chemoresistance in pancreatic tumors. Although the majority of cancer cells may be diminished by chemotherapy, remaining CSC would be able to recapitulate the tumor after treatment cessation and thus cause tumor refraction. For successful treatment, it is required that the CSC population is completely depleted, and as such therapeutic interventions that are able to overcome resistance in CSC should be beneficial.

### 7.2. EMT and Metastasis in Pancreatic Cancer Stem Cells

Several lines of evidence suggest that pancreatic CSC have a high capacity to metastasize. Histological analysis identified rare disseminating CD133^+^ cells at the invasive front of pancreatic tumors [154]. A pancreatic cancer cell line subpopulation of highly migratory cancer cells that exhibited elevated expression of the EMT-related genes Slug and Snail was also characterized by upregulation of the CSC marker CD133 [269]. Interestingly, CD133 itself is important for migration and invasion of this population [269]. In addition, expression of CXCR4 is essential for CD133^+^ cells to maintain their enhanced migratory potential [154].

Pancreatic CSC marked by ALDH expression or CD44- and CD24-positivity also exhibit mesenchymal features, such as reduced E-cadherin expression, and increased expression of Snail and vimentin [151,160]. In a genetically engineered mouse model disseminating pancreatic cancer cells are enriched for CD44^+^CD24^+^ CSC compared to the primary PanIN lesion or tumor, and have increased proliferation rates and spheroid formation capacity *in vitro* [9]. Moreover, it was found that in certain cases metastatic lesions, which are likely established by a cancer cell that underwent EMT, are ALDH positive, whereas the primary pancreatic tumor from the same patient was classified ALDH negative [160]. In addition, ALDH positive pancreatic tumors are generally larger than ALDH negative tumors, poorly differentiated, and associated with a worse prognosis [160].

The positive relationship between EMT and CSC properties could mean that a higher proportion of CSC has the ability to disseminate, though the migratory capacity may not be exclusive to CSC but could also occur in normal cancer cells that undergo EMT. However, CSC may not only disseminate more readily, but would also have the enhanced capability to establish a tumor at a secondary site. A combination of these two properties would make CSC a very competent mediator of metastases formation.

### 7.3. Cancer Stem Cells Generated via EMT

An important issue regarding CSC is the origin of these multipotent cells. Since CSC are very similar to non-malignant stem cells, it is suggested that CSC can be created by oncogenic transformation of tissue-specific stem cells [270,271]. However, in it is still unclear whether an adult stem cell pool resides in the pancreas [272], posing the possibility that there may be an alternative source for pancreatic CSC. Besides, evidence indicates that CSC are not necessarily derived from normal stem cells. Studies in leukaemia [273,274,275] and glioma [276,277] suggest that cancer can arise from progenitor cells or even differentiated cells that have regained the capability of self-renewal.

Since activity of developmental pathways is often involved in maintenance of stemness and the capacity to undergo self-renewal, it is not surprising that activation of these pathways endows pancreatic cancer cells with features indicative of undifferentiated cells, such as the expression of stem-cell markers [168,192,249]. In several types of cancer, poorly differentiated tumors show expression of genes normally found in embryonic stem cells, including targets of Nanog, OCT-4, SOX2 and c-MYC [278].

An interesting possibility is that EMT could contribute to the establishment of CSC via implementation of stem cell-like features. Poor tumor differentiation due to extensive EMT may therefore predispose cells to becoming CSC. Although expression of genes associated with embryonic stem cells in poorly differentiated breast tumors was not restricted to, or related with, the CD44^+^CD24^−^ CSC population [278], it is possible that additional mutations distinguish CSC from non-CSC. Interestingly though, CD24, a marker of pancreatic CSC [149], is highly expressed in embryonic stem cells [279], and could therefore be a stem cell marker whose expression is restricted to CSC even in overall poorly differentiated tumors.

In breast cancer, EMT results in acquisition of CSC properties such as increased spheroid formation and tumorigenicity, indicating that in this tumor type EMT can indeed stimulate generation of CSC [280]. The direct effect of EMT on pancreatic CSC generation remains to be examined, however it was shown that side population cells from pancreatic cancer cell lines, which are enriched for CSC, undergo EMT much more readily than non-side population cells [281]. It is possible that activity of pathways involved in both maintenance of stemness and EMT, such as the developmental pathways described above, could render CSC more prone to undergoing EMT, and conversely may predispose cells that undergo EMT to becoming CSC.

### 7.4. Tumor Micro-Environment Promotes EMT in Pancreatic Cancer

As described earlier, there are many indications that the local micro-environment has a profound effect on pancreatic tumor behavior with respect to growth, invasion, and migration. Here, the possibility that these effects are the result of induction of an epithelial-to-mesenchymal transition is discussed.

Factors secreted by stellate cells stimulate proliferation of pancreatic cancer cells and promote tumor formation upon transplantation in nude mice [104,122], which is consistent with the effects of enrichment for CSC in the cell population. It is possible that PSC enhance tumor establishment via EMT and implementation of stemness in cancer cells, although the requirement of an EMT to mediate the effect of these factors was not investigated. However, co-culturing of pancreatic cancer cells with PSC induced a fibroblast-like morphology, increased expression of Snail and vimentin, decreased E-cadherin levels, and enhanced migration, all indicative of an EMT [282]. Likewise, inflammatory cells and fibroblasts induce an EMT, thereby enhancing the metastatic potential of pancreatic cancer cells [9,259]. The stimulation of an EMT by stromal cells is likely to play a predominant role in the effect that the tumor micro-environment has on the invasive and migratory potential of pancreatic cancer cells. Interestingly, the promotion of EMT by fibroblasts was mediated by growth factors signaling via c-MET [259], a receptor which was also characterized as a pancreatic CSC marker. Another study identified c-MET-, CD44-, and α6β4-receptors as mediators of a tumor matrix triggered increase in migratory potential of pancreatic tumor cells [283]. Especially the CD44 variant isoform 6 appears to be important for coordinating motility in response to signaling by the extracellular matrix [283].

Limited oxygen availability also contributes to EMT in pancreatic cancer cells. Exposure to hypoxic conditions led to a more fibroblast-like phenotype, nuclear translocation of Snail, and loss of E-cadherin, accompanied by increased invasion and migration [284]. The hypoxia-induced EMT was found to be dependent on generation of reactive oxygen species leading to early inactivation of GSK-3β, resulting in activation of the WNT signaling pathway [284]. The observed increase in invasive and migratory potential was however dependent on VEGF and hypoxia inducible factor-1α (HIF-1α) mediated signaling [284]. In pancreatic tumor samples, the expression of HIF-1α was correlated with upregulation of c-MET and its ligand hepatocyte growth factor (HGF) in both pancreatic cancer cells and stromal cells, and is linked with an increase in lymph node metastases [285]. Another study found that under normoxic conditions, expression of the EMT marker Twist is absent or very weak [180]. However, Twist expression is increased as a result of hypoxia, possibly via regulation by HIF-1α [180]. These studies imply that the overall poor vascularization of pancreatic tumors [138] may thus contribute to induction of an EMT.

### 7.5. Contribution of EMT to Give Rise to Stromal Cells

Since cells that undergo EMT adopt a fibroblast-like morphology, it would be possible that tumor associated PSC and fibroblast arise from pancreatic epithelial cells that acquire a mesenchymal phenotype. Indeed, several studies in various tissues indicate that besides the recruitment of mesenchymal cells from the bone marrow [286], and genetic alteration or cytokine stimulation of normal fibroblasts [287,288,289], stromal cells can be formed locally as a result of EMT [290,291,292]. It was shown that activation of the Hedgehog signaling pathway, which facilitates EMT, induces stromal expansion in models of pancreatic cancer [116,293].

In breast cancer, there is evidence that non-malignant stromal cells can be derived from tumor cells via EMT [294]. This is further supported by the finding that both cell types frequently harbor a number of identical genetic alterations [295], including mutations in the *TP53* gene [296]. In this scenario, CSC would be a suitable candidate as cells of origin for tumor stroma, as there appears to be a close connection between this subpopulation of cancer cells and EMT. However, analysis of pancreatic tumors showed that *TP53* was mutated in 61% of the tumors, but this alteration was never found in the associated stroma [297]. This indicates that the pancreatic stromal cells may be derived from the epithelium via EMT, or originate from mesenchymal cells or local fibroblasts, but that pancreatic tumor cells are not likely a source for cancer associated fibroblasts or PSC.

## 8. Conclusions

### 8.1. Re-evaluating the Cancer Stem Cell Concept

The definition of ‘cancer stem cells’ should probably not be taken too strictly. CSC may not be the only cells that can initiate a tumor or replenish the cancer cell population. In fact, some tumors, especially those that have a more differentiated phenotype, may not contain any CSC at all. In others, the acquisition of stem-like features might be a secondary event that takes place after the tumor is formed. However, establishment and maintenance of certain other tumors may indeed rely on CSC, originating from tissue specific stem cells via malignant transformation, or generated from more differentiated cells by oncogenic mutations and the gain of stem-cell like properties.

The identification of CSC remains a challenging issue that relies on techniques that are not free from experimental artifacts. For example, in order to sort cells by FACS, tissues need to be completely dissociated, which may affect the expression of cell surface proteins, including CSC markers. For identification of a CSC subpopulation by dye exclusion, cells are treated with Hoechst 33342 [162]. Those cells that are unable to extrude the dye may experience toxic effects, which could bias their subsequent ability to form spheroids or initiate tumors upon transplantation. Furthermore, immunodeficient mice are generally used to examine *in vivo* tumorigenicity. Since cells of the immune system compose an important part of the tumor micro-environment, implantation of cells in immunocompromised animals may not represent the normal interactions between cancer cells and tumor associated immune cells, which are believed to support pancreatic cancer development.

Although each method is associated with certain drawbacks, together they can be used to gain useful and important insight into the properties of CSC and their function within the tumor. A combination of techniques has identified subpopulations of pancreatic cancer cells that have increased potential to initiate tumors and can give rise to a heterogeneous population of cells, and are thus classified as cancer stem cells. Surface proteins are used as pancreatic CSC markers, but these are not necessarily overlapping in expression [159,160,161], demonstrating that each of the markers used to date is not expressed on the complete CSC population. This may be due to the limitations of functional assays to test the validity of these CSC markers, and the resulting possibility that the employed surface antigens are not per definition expressed by CSC or that expression is not restricted to CSC. More likely, the employed markers are expressed on a higher proportion of CSC compared to other cancer cells, and isolation of cells that express these markers thus results in enrichment for CSC.

Notably, the existence of apparently ALDH negative [159,160], CD133 negative [155], or CD44 negative pancreatic tumors [157] could have several explanations. The first possibility is that the CSC marker negative tumors are not reliant on a specific subset of CSC for their establishment and maintenance. It is likely that adoption of stem-like features is required for certain cells to become tumorigenic, but that does not rule out that other cancer cells may rely on different intracellular changes for their tumorigenicity, and as such acquisition of stem-like features is not essential for these cells to form and maintain tumors.

Another explanation is that the fraction of CSC in the tumor is too low to be detected. Possibly, the aggressiveness of the tumor is related to the fraction of CSC in the population, as these are generally more tumorigenic than non-CSC and are more capable of disseminating. A relation between CSC fraction and survival was established for breast cancer, glioma, and rectal cancer [298,299,300]. In pancreatic tumors, a correlation was found between ALDH positivity and prognosis [160], but the link with CSC proportion has not been examined thus far.

A third option is that the CSC in these tumors are not marked by the specific surface antigen whose expression was examined. CSC marker expression may depend on the signaling pathways on which a cell relies for stemness and could thus vary between CSC. The expression of different markers by pancreatic CSC is supported by the presence of non-overlapping populations of CD133, CD44 and CD24, c-MET, and ALDH expressing cells with CSC properties [159,160,161]. As such, it may be impossible to identify a universal marker of pancreatic CSC due to the heterogeneity of this population.

It is even questionable if ‘the cancer stem cell population’ is truly a separate entity that resides in pancreatic tumors. An alternative possibility is that tumors consist of a heterogeneous collection of cells that exhibit varying degrees of differentiation, depending on the combination of stemness-versus differentiation related genes that are expressed in each cancer cell. This would mean that identification of a CSC- and non-CSC population is not so straightforward, as there may be an intermediate population that is not completely stem-like, but also not fully differentiated.

### 8.2. Inhibition of Stemness as a Treatment Regimen

The extensive stromal reaction that is characteristic of pancreatic tumors can promote an EMT, which is associated with activation of developmental pathways. In turn, the occurrence of an EMT, resulting from stimulation by the micro-environment or genetic alterations in genes that encode components of developmental pathways, may further exacerbate the desmoplastic reaction. In poorly differentiated tumors, cancer cells would overall exhibit more stem-like features. These stem-like cancer cells are enhanced in their ability to disseminate and initiate a tumor, and furthermore harbor increased resistance to chemotherapy, possibly mediated by EMT. The enhanced metastatic potential and reduced sensitivity to chemotherapeutics can both contribute to the relationship between poor differentiation and a worse clinical outcome in pancreatic cancer patients [66,78,160].

Specific targeting of stem-like cancer cells could reduce tumor growth, chemoresistance, and metastasis formation. Since the surface antigens that are employed as CSC markers are predominantly expressed on cells with stem-like features and possibly play a role in maintenance of stemness, therapeutics based on these proteins could perhaps be used to target stem-like cancer cells. *In vivo* administration of a c-MET inhibitor indeed prevented the formation of metastases and decreased pancreatic xenograft growth, while combined treatment with gemcitabine completely stalled tumor growth [161]. Also, pretreatment of pancreatic cancer cells with T-cell mediated lysis inducing antibodies against ESA reduced *in vitro* spheroid formation and tumor initiation upon transplantation in immunocompromised mice [301]. More importantly, *in vivo* treatment of established pancreatic xenografts with this antibody stalled tumor growth, though the anti-tumor effect was not augmented by simultaneous treatment with gemcitabine [301].

Other factors that maintain poor differentiation in pancreatic tumors could potentially also be inhibited for treatment purposes. For example, reversal of EMT by targeting molecular markers of mesenchymal cells may alter the aggressive behavior of pancreatic tumors. In line with this hypothesis, the inhibition of ZEB1 reverted EMT and restored drug sensitivity in pancreatic cancer cells lines [263]. Likewise, treatment with natural agents that reduce mesenchymal morphology and implement an epithelial phenotype increased the sensitivity of pancreatic cancer cells to gemcitabine [264].

Another possibility to inhibit stemness in cancer cells is by targeting developmental pathways such as the Hedgehog, NOTCH, WNT, and Nodal/Activin pathways, which are frequently active in pancreatic tumors [187,202,239,254]. Preclinical studies support the hypothesis that inhibition of these pathways can reduce stemness, which results in decreased tumorigenicity and migratory capacity, as well as restored sensitivity to chemotherapeutics. For instance, blocking of NOTCH signaling with GSI-18 repressed the expression of the CSC marker ALDH, rendered pancreatic cancer cells anchorage-dependent for proliferation and inhibited tumor initiation in mice [239]. Treatment with the ALK4/7 inhibitor SB431542, which blocks Nodal/Activin signaling, reduced spheroid formation and invasion of pancreatic cancer cells *in vitro* [254]. Pretreatment with a combination of the Nodal/Activin pathway inhibitor and gemcitabine irreversibly depleted cells expressing the CSC marker CD133, and prevented xenograft formation in recipient mice [254]. In pre-established xenograft tumors, treatment with the Nodal/Activin pathway inhibitor, in combination with gemcitabine and a Hedgehog inhibitor to improve drug delivery, decreased tumor growth leading to long-term stable disease [254]. This effect was accompanied by a reduction in stem-like features and evidence of differentiation in the xenograft tumor [254].

Also, inhibition of the Hedgehog pathway by cyclopamine or vismodegib was shown to revert EMT, reduce CSC characteristics, induce apoptosis, and restore sensitivity to gemcitabine treatment *in vitro*. Moreover, suppression of Hedgehog signaling downregulated CSC marker expression, mediated tumor regression, and diminished metastatic spread in mouse models [118,191,260,302]. However, despite these promising data a phase I clinical trial showed that vismodegib was hardly effective for the treatment of patients with advanced pancreatic tumors, possibly because these tumors are less dependent on interplay of Hedgehog components between stromal and cancer cells than tumors in an earlier disease stage [303].

Since crosstalk between stromal cells and pancreatic cancer cells can contribute to activation of stemness related pathways, modulation of the tumor micro-environment could have beneficial effects in pancreatic cancer patients. As such, injection of genetically modified mesenchymal stem cells expressing a therapeutic gene, which are recruited to the tumor associated stroma, decreased tumor volume in a mouse model of pancreatic cancer [304]. Targeting specific stromal components, for example by inhibiting fibroblast activation protein (FAP), which was previously shown to reduce stromatogenesis in KRAS driven tumors of different tissues [305], may also reduce the stimulatory effect of the micro-environment on pancreatic tumors.

**Figure 1:**
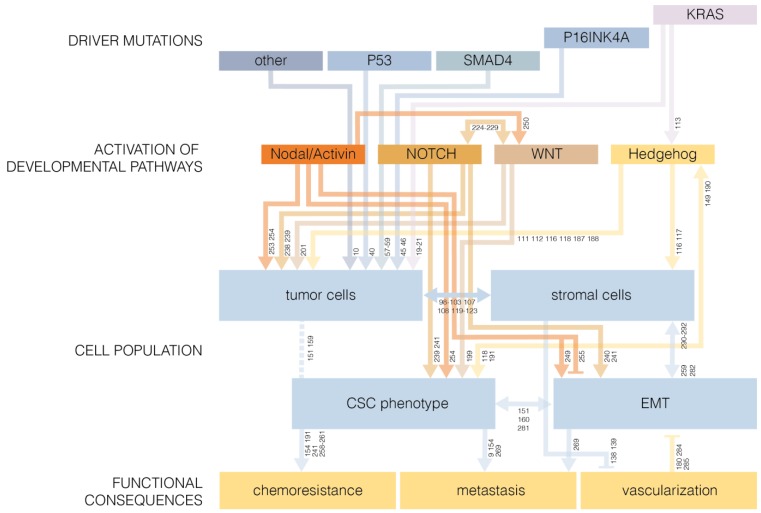
Flow-chart of the activating or inhibiting interactions between events that could mediate the transition towards cancer stem cells and EMT, as well as their functional consequences. The dotted line indicates that tumor cells can adopt a more CSC-like phenotype, rather than to induce CSC as a separate entity. Reference numbers of studies supporting each interaction are shown.

CSC-like features, EMT, and activation of developmental pathways in pancreatic cancer cells should not be viewed as isolated events (Figure 1). Not only do these features often occur simultaneously, but they also appear to reinforce each other, possibly because they are all related to the expression of genes associated with stemness. Perturbation of these events may induce a transition from a poorly differentiated, aggressive tumor to a tumor with a more differentiated phenotype that is generally associated with a better prognosis. Therapeutic agents aimed at the inhibition of stemness via an approach based on CSC- or EMT markers, developmental signaling, or tumor micro-environment that show promising results in pre-clinical studies could prove beneficial for the treatment of pancreatic cancer patients.
